# A Multidomain Model for Dementia Classification using Harmonized LASI and LASI-DAD Data

**DOI:** 10.64898/2026.06.13.26354833

**Published:** 2026-06-24

**Authors:** Shweta Anand, Krishna P. Miyapuram

**Affiliations:** 1 Cognitive Science, Indian Institute of Technology Gandhinagar, Palaj, Gandhinagar 382055, Gujarat, India

**Keywords:** dementia, classification, multidomain modelling, machine learning, interpretability, older adults, India, LASI-DAD

## Abstract

Dementia classification in heterogeneous populations is complicated by the influence of education, language, socioeconomic position and health status on cognitive test performance. Approaches that rely on fixed cognitive thresholds or isolated predictor sets may therefore perform inconsistently across diverse older adult populations.

We developed and internally validated a multidomain classification model using harmonized data from the Longitudinal Ageing Study in India (LASI) and its diagnostic sub-study, LASI-DAD. Clinical dementia status was defined as a binary outcome derived from consensus-based Clinical Dementia Rating (CDR) assessments, averaged across 20 multiply imputed outcome datasets and finalised using a 0.5 threshold. The analytic sample comprised 3,186 participants after exclusion of those with mild cognitive impairment. Twenty-two predictors spanning cognitive performance, informant-reported decline, cardiometabolic biomarkers and sociodemographic characteristics were retained. Missing predictor values were addressed using k-nearest neighbours imputation. Model development used a stratified 70:30 train-test split, with nested cross-validation conducted within the training set only, and class imbalance corrected using the Synthetic Minority Oversampling Technique (SMOTE) applied exclusively within training folds. Five supervised learning approaches were evaluated: logistic regression, random forest, gradient boosting, XGBoost and support vector machines.

The final logistic regression model achieved an area under the receiver operating characteristic curve (ROC-AUC) of 0.932 and an average precision of 0.668 on the held-out set. At the optimal probability threshold of 0.70, sensitivity was 0.771, specificity was 0.905, positive predictive value was 0.325 and negative predictive value was 0.985. A cognition-only comparator, restricted to task-based cognitive measures and run through the same pipeline, yielded a ROC-AUC of 0.908 and average precision of 0.620, indicating incremental discriminatory value from the full multidomain feature set. Dementia prevalence increased progressively across model-derived risk strata, reaching approximately 50% in the highest category. Permutation importance and SHAP analyses identified informant-reported decline and orientation as the strongest contributors to classification, with cardiometabolic variables providing smaller but consistent incremental contributions.

Dementia classification in a socially and clinically heterogeneous Indian cohort can be improved by integrating cognitive, informant, cardiometabolic and sociodemographic information within a single interpretable model. The strongest predictive signal was carried by cognitive and informant measures, with non-cognitive features adding structure around that core. The model requires external validation and calibration before broader application can be considered.

## 1. INTRODUCTION

Dementia classification remains difficult in ageing populations where cognitive performance is shaped by more than neurodegenerative change alone. Educational attainment, literacy, language background, health status and access to formal assessment all influence how individuals perform on cognitive tests, making direct interpretation across heterogeneous populations inherently unstable (Ardila, 2005; Manly, 2006; Stern, 2009). This challenge is especially acute in low- and middle-income settings, where social and clinical variation is often substantial and where dependence on any single screening measure may contribute systematically to both under recognition and misclassification of dementia (Prince et al., 2015; World Health Organization, 2021).

The deeper problem is not simply that cognitive tests perform imperfectly across diverse populations. Dementia-related decline unfolds across multiple biological and functional domains simultaneously, and these processes rarely operate in isolation. Vascular and metabolic dysregulation, systemic inflammation and functional deterioration as reported by a close informant all reflect aspects of the dementia syndrome that task-based cognitive assessment alone may not fully capture, particularly in populations where low scores may reflect educational disadvantage as much as neurodegeneration (Jorm, 2004; Whitmer et al., 2005; Walker et al., 2019). An approach that integrates information across these domains is better positioned to represent that complexity than one anchored to a single score or threshold.

Predictive modelling offers a natural framework for handling such multidimensional clinical structure, particularly where predictors are correlated, class imbalance is present and measurement involves uncertainty. Machine learning methods can accommodate complex feature combinations and, under appropriate conditions, yield stronger discrimination than conventional single-domain approaches (Deo, 2015). That discriminatory gain, however, is of limited value in clinical or public health settings unless model behaviour can be understood and communicated clearly. There is consequently growing methodological interest in approaches that combine predictive strength with transparent structure, where the contribution of individual features can be examined and interpreted rather than simply reported (Rudin, 2019; Lundberg and Lee, 2017).

These considerations are particularly salient in India, where LASI and its linked diagnostic sub-study, LASI-DAD, provide a rare opportunity to examine dementia within a nationally representative population marked by substantial heterogeneity in education, language, comorbidity and access to care (Arokiasamy et al., 2012; IIPS et al., 2020; Lee et al., 2020). National estimates derived from these data suggest that dementia burden in India considerably exceeds earlier regional projections and may affect several million older adults (Lee et al., 2023). That scale makes accurate classification both clinically urgent and analytically demanding.

Despite the richness of LASI-DAD, relatively little published work has developed interpretable multidomain classification models anchored to clinically derived dementia outcomes within this setting. Most analyses have focused on prevalence estimation and descriptive characterization rather than on predictive modelling with explicit attention to feature interpretability. The novelty of the present study lies not in using LASI-DAD for dementia research per se, but in building and internally validating an interpretable multidomain classification model anchored to clinically derived CDR based outcomes within this population. This matters because large population based datasets of this kind inevitably involve missingness, correlated predictors and measurement variability, all of which complicate both model development and interpretation. A further complication concerns the outcome itself. The CDR is a clinician-rated global staging instrument used to classify dementia severity. In large population datasets, CDR based dementia assignment is not always fully observed, and the LASI-DAD protocol accordingly provides 20 multiply imputed versions of the dementia outcome to reflect residual diagnostic uncertainty. A classification model developed in this setting must therefore accommodate imprecision in the outcome as well as complexity across predictors.

The present study develops and internally validates a multidomain classification model for dementia using harmonized LASI and LASI-DAD data. Predictors were drawn from cognitive performance, informant-reported decline, cardiometabolic biomarkers and sociodemographic characteristics. Multiple supervised learning approaches were compared within a nested cross-validation framework with class imbalance correction, and the final model was examined using permutation importance and SHapley Additive exPlanations (SHAP) to characterize the contribution of individual features. The central question is whether integrating information across these domains improves dementia classification beyond what cognitive measures alone provide, and whether such performance can be achieved with a model whose behaviour remains interpretable.

## 2. METHODOLOGY

### 2.1 Data Source and Study Population

Data were drawn from LASI and LASI-DAD and harmonized across component files using the unique participant identifier *prim_key*. LASI is a nationally representative longitudinal survey of adults aged 45 years and older in India, covering health, economic and social dimensions of ageing (Arokiasamy et al., 2012; IIPS et al., 2020). LASI-DAD is a population based diagnostic sub-study embedded within LASI, designed to provide rigorous clinical assessment of cognitive status and dementia in a representative subsample of older LASI participants aged 60 and above, using neuropsychological testing, informant interview and consensus-based clinical review (Lee et al., 2020). Linkage of the two datasets provided cognitive, informant-reported, biomarker and sociodemographic information for participants who had undergone detailed cognitive assessment within LASI-DAD.

The analytic sample was restricted to participants for whom the merged files supported supervised binary classification. Individuals classified as having mild cognitive impairment (MCI) were excluded so that the classification boundary was defined between dementia and cognitively unimpaired status, reducing diagnostic ambiguity at the boundary. The model developed here should therefore be interpreted as a dementia classification model rather than a prodromal detection model; the exclusion of MCI means that the analytic task is distinguishing established dementia from cognitively unimpaired status rather than identifying subtle or early-stage impairment. The final analytic sample comprised 3,186 participants. Although probability based survey weights are available within LASI, they were not applied because the objective was predictive modelling rather than population-level prevalence estimation; weighting optimises representativeness for descriptive inference but it is not required, and may introduce distortions, in supervised classification (Deo, 2015).

### 2.2 Outcome Definition

The study outcome was a binary dementia classification derived from the LASI-DAD dementia imputation files. The underlying clinical process involved consensus based CDR assessment conducted by trained clinicians, producing a global CDR score that reflects severity of cognitive and functional impairment across six domains. For the purpose of supervised classification, a binary dementia indicator was constructed from these assessments. Because CDR based dementia assignment was not fully observed with certainty for every participant, the LASI-DAD protocol provides 20 multiply imputed versions of the binary dementia indicator (Lee et al., 2025). For each participant, these 20 imputed values were averaged to produce a continuous value reflecting the degree of agreement across imputations. A 0.5 threshold was then applied to this averaged value to assign the final binary training label, consistent with standard practice for handling multiply imputed binary outcomes in predictive modelling (Sterne et al., 2009). This approach ensured that outcome uncertainty was partially retained in the modelling framework rather than resolved arbitrarily through a single imputed dataset. Participants whose averaged dementia probability fell near the 0.5 boundary represent a source of residual outcome uncertainty; formal sensitivity analysis examining model performance after excluding such cases represents an important direction for future work.

### 2.3 Predictors

Predictors were selected to reflect the multidomain structure of dementia related classification while remaining anchored to variables with sufficient completeness and construct validity for model development. The final predictor set comprised 22 variables spanning four domains.

Cognitive performance was represented by MMSE orientation score, immediate word recall, delayed word recall, story recall, clock drawing, animal fluency, and two composite scores: a memory composite derived from word and story recall tasks, and an executive composite derived from clock drawing and animal fluency. These composites were retained alongside their component tasks because they capture aggregate domain-level signal that may not be fully represented by any single subtest. The inclusion of both composites and their component tasks introduces collinearity within the cognitive domain; while this does not impair logistic regression’s discriminatory performance, individual coefficient estimates for correlated cognitive predictors should be interpreted with caution, and permutation importance and SHAP values for these features reflect shared rather than wholly independent contributions. Informant-reported decline was captured through the Informant Questionnaire on Cognitive Decline in the Elderly (IQCODE), a validated instrument that assesses change in everyday cognitive functioning as reported by a close informant (Jorm, 2004). Cardiometabolic biomarkers included glycated haemoglobin (HbA1c), total cholesterol, HDL cholesterol, LDL cholesterol, triglycerides, high-sensitivity C-reactive protein (hsCRP), homocysteine, vitamin B12, 25-hydroxy vitamin D and albumin. Sociodemographic characteristics comprised age, sex and educational attainment.

The Hindi Mental State Examination (HMSE), although available within LASI-DAD, was not included in the predictor set. Linguistic familiarity with Hindi is not uniformly distributed across India’s population, and performance on the HMSE may therefore reflect regional language background as much as cognitive status in participants whose primary language is not Hindi. Including it risked introducing systematic measurement inequivalence across subpopulations, which would undermine the generalizability of the model across India’s linguistically diverse regions.

A full list of predictors by domain is provided in Supplementary Table S1.

### 2.4 Missing Data

Missingness across the retained predictors was low to moderate and is summarized in Supplementary Figure S3. Missing predictor values were imputed using k-nearest neighbours (KNN) imputation with k=5, selected because it preserves local data structure without parametric assumptions about predictor distributions. Categorical variables were ordinally encoded prior to imputation to enable distance based neighbour computation. All continuous predictors were standardized to zero mean and unit variance prior to model fitting. Imputation, standardization and oversampling were fitted exclusively within the training pipeline; no information from the validation or held-out test data was used during preprocessing, preventing any form of data leakage.

### 2.5 Model Development and Evaluation

The dataset was partitioned using a stratified 70:30 train-test split so that class proportions were preserved across training and held-out sets. The held-out test set was withheld entirely from model development and used exclusively for final performance evaluation.

Nested cross-validation was conducted only within the 70% training set. The inner loop comprised three folds used for hyperparameter optimization, with tuning performed by maximising ROC-AUC. The outer loop comprised five folds used to estimate model performance during development and to reduce optimism relative to a single train-validation split. After model selection, the final pipeline was refit on the complete training set and evaluated once on the held-out test set.

Because dementia cases represented approximately 13.6% of the analytic sample, class imbalance was addressed within training folds using SMOTE, which generates synthetic minority-class observations by interpolating between existing cases (Chawla et al., 2002). SMOTE was applied only within training folds to prevent synthetic cases from appearing in validation or test data.

Five supervised learning approaches were evaluated - logistic regression, random forest, gradient boosting, XGBoost and support vector machines (SVM). Final model selection was based on held-out test set discrimination, consistency between cross-validation and test-set performance, and compatibility with transparent post-hoc interpretation. Although several ensemble methods showed competitive cross-validation performance, logistic regression achieved the strongest balance on the held-out test set and was retained as the final model.

A cognition-only comparator model was developed using the same pipeline, restricted to the eight task-based cognitive predictors and excluding IQCODE, cardiometabolic biomarkers and sociodemographic variables. Sociodemographic variables were excluded from the comparator to provide a conservative test of the incremental value of non-cognitive domains specifically. This comparator quantified the discriminatory value of the full multidomain predictor set beyond cognitive task performance alone.

Performance was assessed using ROC-AUC and average precision. Average precision was prioritized alongside ROC-AUC because it is more sensitive to model performance on the minority class under imbalanced outcome distributions (Saito and Rehmsmeier, 2015). Sensitivity, specificity, positive predictive value (PPV), negative predictive value (NPV) and F1 score were evaluated at the optimal probability threshold determined using Youden’s index. Predicted probabilities from the final model were retained as continuous classification scores and subsequently grouped into five ordered risk categories (very low, low, moderate, high, very high), defined by equal-width probability intervals across the 0 to 1 score range, to examine how observed dementia prevalence varied across the score distribution.

### 2.6 Model Interpretability

Model interpretability was examined using two complementary approaches. Permutation feature importance was computed on the held-out test set by measuring the reduction in ROC-AUC following random permutation of each predictor in turn, with results averaged across multiple permutations to reduce sampling variability. SHAP was used to decompose individual model predictions into additive feature contributions, allowing both the magnitude and direction of each predictor’s influence to be assessed at the level of individual observations and summarized across the test set (Lundberg and Lee, 2017). All analyses were conducted in Python within a reproducible modelling pipeline.

## 3. RESULTS

### 3.1 Sample Characteristics

The analytic sample comprised 3,186 participants, of whom 433 (13.6%) were classified as having dementia. The sample had a mean age of 73.88 years (SD 8.72), was slightly majority female (52.9%), and was predominantly rural (62.8%). A substantial proportion had no formal education (32.7%), reflecting the educational heterogeneity characteristic of older cohorts in India. Full sample characteristics are presented in Table 1.

Exploratory analysis indicated that participants classified as having dementia tended to be older and performed markedly worse across cognitive and informant based measures. Differences across cardiometabolic variables were present but less pronounced. Within the cognitive variables, correlation structure showed expected clustering across memory-related measures, while informant-reported decline aligned more strongly with clinical dementia status than with any single cognitive subtest.

Lower-dimensional projections of the feature space confirmed that dementia and non-dementia cases were not cleanly separated along any single axis, but occupied overlapping regions defined by combinations of variables across domains.

### 3.2 Model Discrimination

**Figure 1.**
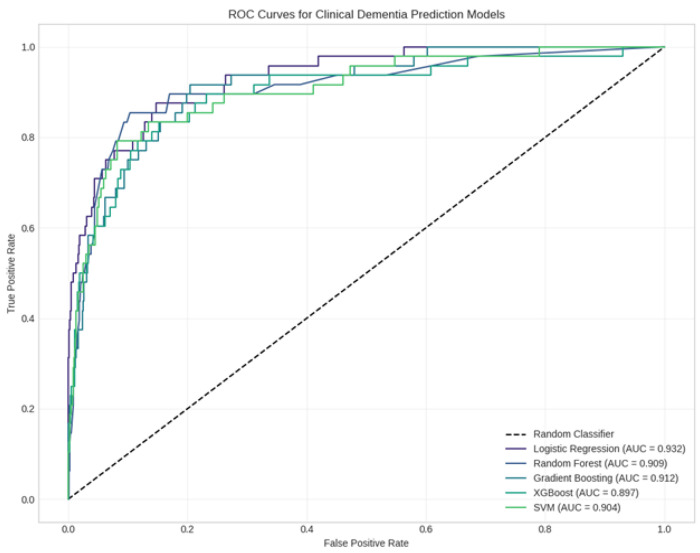
Receiver operating characteristic curves for candidate dementia classification models on the held-out test set.

Performance on the held-out test set showed clear differences across candidate models. The final logistic regression model achieved a ROC-AUC of 0.932, exceeding random forest (0.909), gradient boosting (0.912), XGBoost (0.897) and SVM (0.904). Across the full operating range, logistic regression maintained the strongest overall discrimination and did so with greater consistency between cross-validation and test-set performance than the ensemble methods. Comparative performance across candidate models is presented in Table 2, and ROC curves are shown in Figure 1.

Because the outcome distribution was imbalanced, performance was also examined using average precision. The logistic regression model achieved an average precision of 0.668, again exceeding all alternative models. Precision-recall curves are presented in Supplementary Figure S7.

At the optimal probability threshold of 0.70, sensitivity was 0.771 and specificity was 0.905. At this threshold, the model yielded 735 true negatives, 77 false positives, 11 false negatives and 37 true positives, corresponding to a PPV of 0.325, NPV of 0.985 and F1 score of 0.458. The high NPV indicates strong performance in identifying individuals unlikely to have dementia; the more modest PPV reflects the challenge of positive prediction under the prevailing class distribution. The confusion matrix is presented in Supplementary Figure S6. The cognition-only comparator achieved a ROC-AUC of 0.908 and average precision of 0.620, confirming that the full multidomain model provided incremental discriminatory value beyond cognitive measures alone.

### 3.3 Risk Stratification

**Figure 2.**
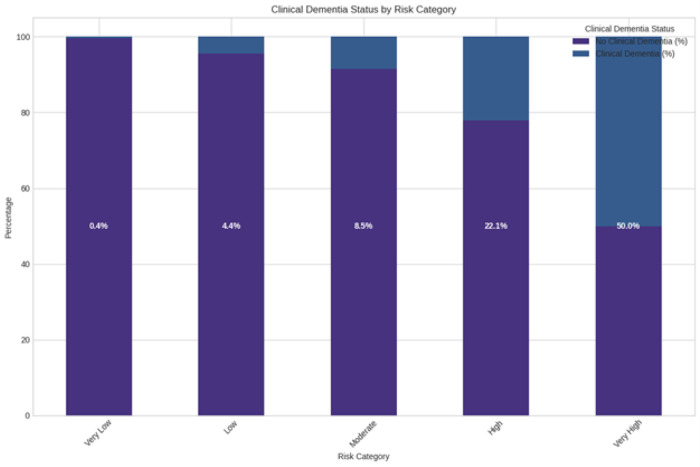
Observed dementia prevalence across model-derived risk categories.

Examination of predicted probabilities showed that model output organized the sample into a graded risk distribution. Dementia cases were concentrated disproportionately within the higher predicted-probability categories, while most non-dementia participants fell within the lower strata. Dementia prevalence increased progressively across the five ordered risk groups, reaching approximately 50% in the highest category. Around half of all dementia cases were concentrated within this upper stratum, despite it representing a relatively small proportion of the overall sample. Observed dementia prevalence by risk stratum is presented in Supplementary Table S2. Risk stratification results are shown in Figure 2, and the broader distribution of model scores is presented in Supplementary Figure S1.

### 3.4 Feature Contribution and Model Structure

**Figure 3.**
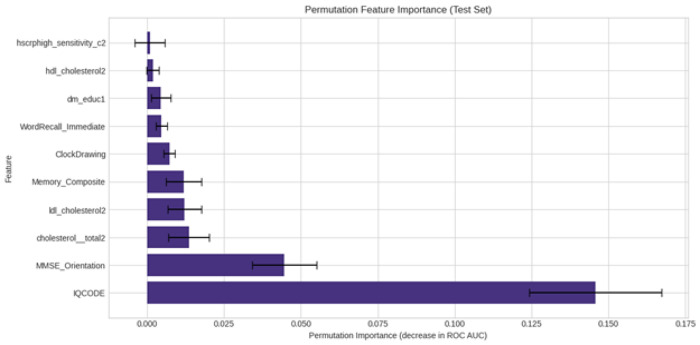
Permutation feature importance on the held-out test set, expressed as mean decrease in ROC-AUC following predictor permutation.

Feature importance analysis indicated that classification was not driven by any single predictor, though contributions were clearly unequal. Permutation importance on the held-out test set showed the largest reductions in ROC-AUC following permutation of IQCODE and MMSE orientation, indicating that these two features carried the strongest individual signal. Composite memory scores, total cholesterol and LDL cholesterol followed, with smaller but non-trivial contributions. The full permutation importance plot is shown in Figure 3.

**Figure 4.**
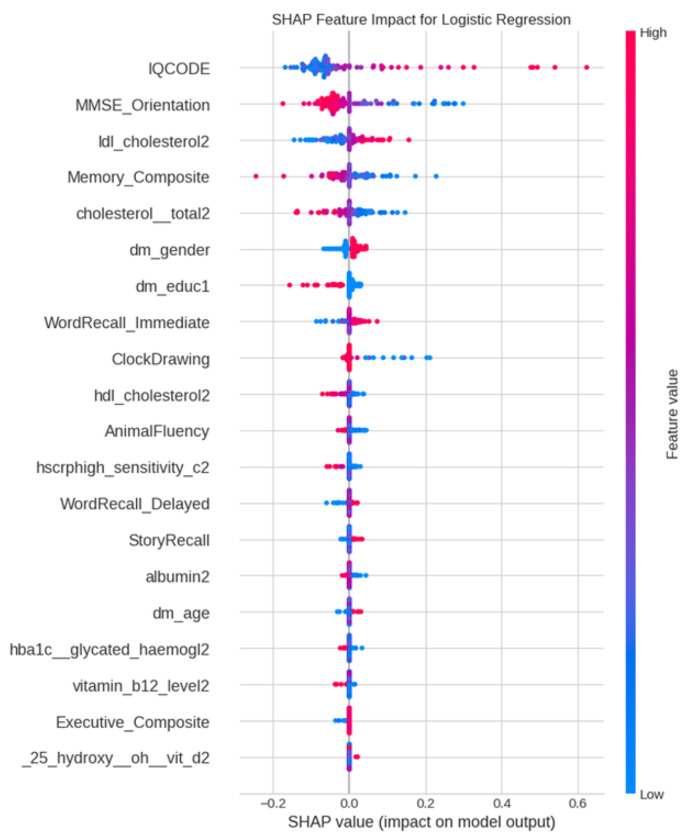
SHAP feature impact summary for the final logistic regression model. Each point represents one observation; colour indicates feature value (red = high, blue = low).

SHAP analysis confirmed and extended this picture. Higher IQCODE scores were consistently associated with increased predicted dementia risk, while preserved orientation and better performance across memory tasks were associated with lower predicted risk. Cardiometabolic variables showed smaller and more variable SHAP contributions, with the direction of their influence dependent on the broader configuration of features present in each individual. The spread of SHAP values indicated that predictor contributions were not fixed in magnitude, but varied with the overall clinical profile of each participant. The SHAP summary plot is shown in Figure 4, and the SHAP feature importance bar plot is presented in Supplementary Figure S2.

Taken together, these findings indicate that classification was anchored primarily by cognitive and informant based measures, with cardiometabolic and demographic variables contributing additional structure around that core signal.

## 4. DISCUSSION

The present findings support a view of dementia classification as inherently multidomain rather than reducible to a single threshold or score. Across an internally validated held-out test set, a logistic regression model integrating cognitive, informant, cardiometabolic and sociodemographic predictors achieved strong discrimination, outperforming both a cognition-only comparator and more complex ensemble approaches. The pattern of feature contributions was broadly coherent with clinical expectations, and the model organized the cohort into a meaningful risk gradient in which observed dementia prevalence increased steadily across higher score strata.

The importance of the informant-reported measure deserves particular attention. IQCODE contributed more strongly to classification than any individual cognitive task, including the orientation subscale of the MMSE. Informant-based instruments capture change in everyday functioning over time rather than performance at a single testing occasion, and are therefore less sensitive to the educational and linguistic factors that can depress task-based scores in populations with limited formal schooling (Jorm, 2004). In the Indian setting, where a substantial proportion of older adults have had little or no formal education, this property may be especially important. A close informant’s account of whether someone has changed, rather than where they currently stand relative to a normative threshold, provides a perspective anchored to the individual’s own baseline rather than to population norms that may not apply equally across the sample. The present results are consistent with earlier work demonstrating the incremental value of IQCODE over cognitive testing in diverse and educationally heterogeneous populations (Jorm, 2004; Prince et al., 2015).

This connects to a broader point about the Indian context. In a country as socially and linguistically varied as India, lower cognitive test scores cannot always be read straightforwardly as evidence of neurodegenerative change. They may equally reflect unequal access to schooling, unfamiliarity with the testing format, or disadvantage on measures that carry implicit cultural assumptions. At the same time, those same individuals may face substantially greater exposure to vascular, metabolic and environmental risks across their lives. The difficulty is therefore not only that many dementia-related variables are correlated, but that they tend to be correlated in socially patterned ways, clustering within the same individuals and communities in a manner that a single-domain model cannot represent. This is consistent with broader evidence that socioeconomic inequalities in dementia risk are partly explained through clusters of modifiable exposures rather than isolated predictors (Deckers et al., 2019; Livingston et al., 2020).

The decision not to include HMSE derived measures in the predictor set also deserves a brief explanation. LASI-DAD made important efforts to contextualize cognitive assessment for the Indian setting, and the HMSE was developed precisely to reduce the dependence on English language in testing. Nevertheless, familiarity with Hindi is itself not uniformly distributed across India, and performance on a Hindi language instrument may differ between a participant from Uttar Pradesh and one from Tamil Nadu for reasons unrelated to cognitive status. Including a variable whose interpretation is confounded by regional linguistic asymmetry would have introduced a source of measurement inequivalence that is difficult to correct for within a single model. Exclusion was therefore a methodological precaution rather than a dismissal of the instrument’s clinical value.

The contribution of cardiometabolic variables, though more modest than that of cognitive and informant measures, warrants comment. Dementia is increasingly understood as shaped not only by neurodegeneration in a narrow sense, but by vascular, metabolic and inflammatory processes that accumulate and interact over time (Biessels and Despa, 2018; Whitmer et al., 2005; Walker et al., 2019). In the present analysis, cardiometabolic features did not dominate the classification boundary, and their individual permutation importance values were small. Their aggregate contribution was nonetheless non-trivial, and directional patterns in the SHAP analysis, including higher hsCRP and dysregulated lipid profiles associated with increased predicted risk, are consistent with the known biology. Given the collinearity introduced by retaining both cognitive composites and their component tasks, precise attribution of importance to individual cardiometabolic features should be interpreted cautiously; what the SHAP values reflect is the marginal contribution of each predictor given the full configuration of features present, not a stable independent effect. These features may become more prominent in models designed to capture earlier or prodromal stages of decline, where the cognitive signal is less dominant.

One of the more consequential findings was that logistic regression matched or exceeded the performance of all ensemble approaches on the held-out test set. This should not be interpreted as a general argument against non-linear methods in dementia research. What it suggests, more specifically, is that in the present dataset the additional flexibility of gradient boosting, random forest or XGBoost did not yield a performance advantage sufficient to outweigh the benefits of consistency and interpretability. In medicine, predictive models are ultimately judged not only by their discrimination but by whether their behaviour can be understood and communicated to those who might use them. A model that discriminates well and can be interrogated through SHAP and permutation importance is more likely to support rather than obstruct clinical reasoning than one that achieves marginally higher AUC at the cost of opacity. The present findings are therefore consistent with the broader methodological position that accuracy and intelligibility need not be treated as mutually exclusive objectives in high-stakes classification settings (Rudin, 2019).

The risk stratification results also deserve direct consideration. The model generated continuous classification scores that organized the cohort into an ordered gradient, with dementia prevalence increasing steadily across risk categories and reaching approximately 50% in the highest stratum. In settings where specialist evaluation cannot be offered universally, such a gradient may be more operationally useful than a binary screen, allowing resources and follow-up to be directed preferentially toward those with the highest predicted scores. The high NPV of 0.985 at the selected threshold indicates strong performance in identifying individuals unlikely to have dementia, which has particular operational relevance for ruling out cases in resource-limited settings. This prioritization function requires, however, that the model’s score outputs are well-calibrated. Calibration was not formally assessed in the present study, and this represents a significant limitation. Until formal calibration is established through calibration curves, Brier score and observed versus expected prevalence by risk decile, the model is best understood as a discrimination and ranking tool rather than a source of reliable individual probability estimates.

Several limitations shape the interpretation of these findings. The model was internally validated within a single linked dataset and was not tested against an independent cohort, meaning that the observed performance may not generalise to other populations or data collection settings. The outcome was constructed by averaging across 20 imputed datasets, which was a reasonable response to the structure of the available data but means the training target was not entirely free of diagnostic uncertainty; formal sensitivity analysis after excluding participants with averaged dementia probability near the 0.5 boundary should be conducted in future work. Although the retained predictor set was intentionally multidomain, a considerably wider range of potentially relevant variables could not be incorporated, including environmental exposures, occupational history, sensory impairment and psychosocial factors identified in the broader risk factor literature (Livingston et al., 2020; Yu et al., 2020); their absence reflects the constraints of the harmonized analytic dataset rather than any judgement about their relevance. The inclusion of both cognitive composites and their component tasks introduces collinearity that, while not impairing discrimination, complicates the interpretation of individual feature contributions; future work should compare models using components only or composites only to assess the sensitivity of feature rankings to this decision. Survey weights were not applied because the aim was predictive modelling rather than prevalence estimation. Finally, the analysis is cross-sectional and cannot establish whether the retained predictors represent early determinants, concurrent correlates or downstream markers of decline.

These limitations mark the natural next steps. External validation in independent cohorts should be a priority, particularly given India’s regional and linguistic diversity. Formal calibration analysis and decision-curve analysis would clarify whether the observed discrimination translates into clinically meaningful utility at realistic decision thresholds. Sensitivity analyses examining model performance after excluding participants with uncertain outcome classification, and comparing component-only and composite-only model variants, would strengthen methodological confidence in the reported feature contributions. There is also a strong case for moving beyond the cross-sectional framework; dementia related risk accumulates over time, and models incorporating longitudinal change in cognitive and functional status may capture progression more faithfully. The subsequent waves of LASI and LASI-DAD provide a natural opportunity to examine whether the present approach retains its performance when applied prospectively and whether individual risk trajectories can be modelled directly.

## 5. CONCLUDING REMARKS

This study shows that dementia classification in a heterogeneous Indian cohort can be approached as a multidomain problem using harmonized LASI and LASI-DAD data. Combining cognitive, informant, cardiometabolic and sociodemographic information within a single logistic regression model yielded strong internal discrimination and a meaningful risk gradient, while retaining sufficient transparency for feature contributions to be examined and interpreted. The predictive signal was driven primarily by informant-reported decline and orientation, with non-cognitive features adding structure around that core. The model is internally validated, cross-sectional and dependent on an imputed outcome; calibration, subgroup evaluation and external validation remain necessary before broader application can be considered. In a population where educational, linguistic and social heterogeneity complicates the interpretation of any single measure, the value of this approach lies in showing that dementia classification can accommodate complexity while remaining interpretable.

## Supplementary Material

1

## Figures and Tables

**Table 1.** Sample characteristics of the analytic cohort

Characteristic	Value
Total sample	3,186
Age, years (mean ± SD)	73.88 ± 8.72
Sex, Female, n (%)	1,684 (52.9)
Sex, Male, n (%)	1,502 (47.1)
No formal education, n (%)	1,042 (32.7)
1-5 years of schooling, n (%)	682 (21.4)
6-9 years of schooling, n (%)	567 (17.8)
10-12 years of schooling, n (%)	491 (15.4)
13+ years of schooling, n (%)	404 (12.7)
Urban, n (%)	1,186 (37.2)
Rural, n (%)	2,000 (62.8)
Dementia, n (%)	433 (13.6)
Non-dementia, n (%)	2,753 (86.4)

**Table 2.** Comparative performance of candidate models on the held-out test set

Model	ROC-AUC
Logistic Regression	0.932
Gradient Boosting	0.912
Random Forest	0.909
SVM	0.904
XGBoost	0.897

**Table 3.** Performance of the final logistic regression model at the optimal probability threshold (0.70)

Metric	Value
ROC-AUC	0.932
Average Precision	0.668
Optimal threshold	0.70
Sensitivity	0.771
Specificity	0.905
Positive predictive value (PPV)	0.325
Negative predictive value (NPV)	0.985
F1 score	0.458
True negatives	735
False positives	77
False negatives	11
True positives	37

## Data Availability

Data from LASI and LASI-DAD are available to registered researchers through the Longitudinal Ageing Study in India data repository at the International Institute for Population Sciences (https://www.iipsindia.ac.in).
