# Supplementary material for: A Multidomain Model for Dementia Classification using Harmonized LASI and LASI-DAD Data
Source: medRxiv. 2026 Jun 24:2026.06.13.26354833. Preprint. [Version 1] doi: 10.64898/2026.06.13.26354833 (PMC13320939; doi:10.64898/2026.06.13.26354833)
Supplement: 1 [file NIHPP2026.06.13.26354833v1-supplement-1.pdf]

medRxiv preprint doi: https://doi.org/10.64898/2026.06.13.26354833; this version posted June 24, 2026. The copyright holder has placed this preprint (which was not certified by peer review) in the Public Domain. It is no longer restricted by copyright. Anyone can legally share, reuse, remix, or adapt this material for any purpose without crediting the original authors.

SUPPLEMENTARY MATERIALS

Supplementary Figure S1.

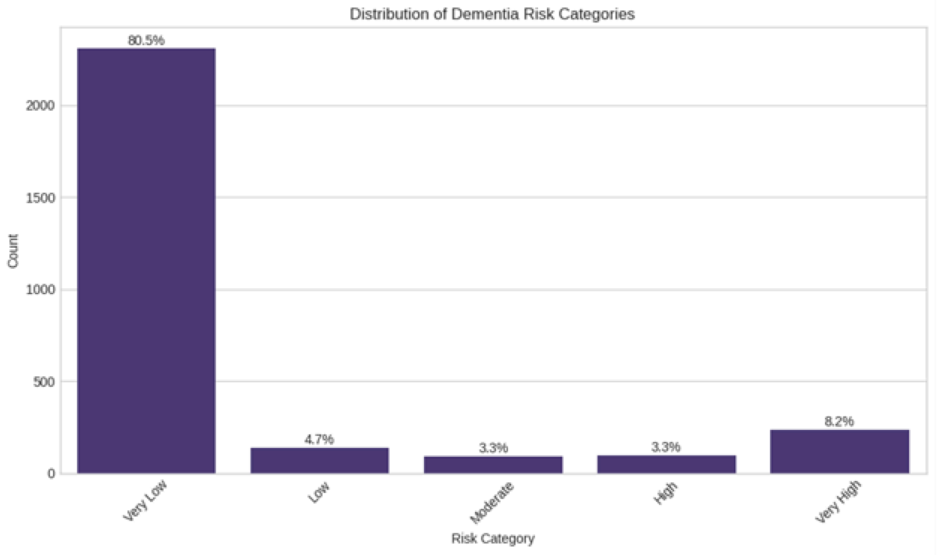

Supplementary Figure S1. Distribution of participants across model-derived dementia risk categories.

Supplementary Figure S2.

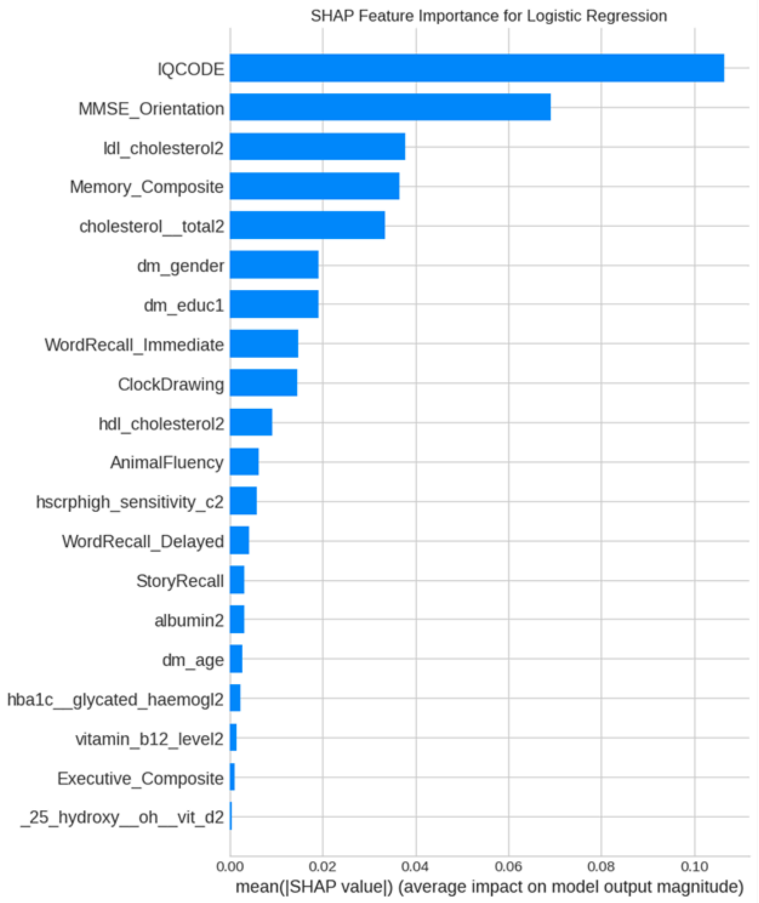

medRxiv preprint doi: https://doi.org/10.64898/2026.06.13.26354833; this version posted June 24, 2026. The copyright holder has placed this preprint (which was not certified by peer review) in the Public Domain. It is no longer restricted by copyright. Anyone can legally share, reuse, remix, or adapt this material for any purpose without crediting the original authors.

Supplementary Figure S2. Mean absolute SHAP values for the final logistic regression model, summarizing average feature contribution magnitude across the test set.

Supplementary Figure S3.

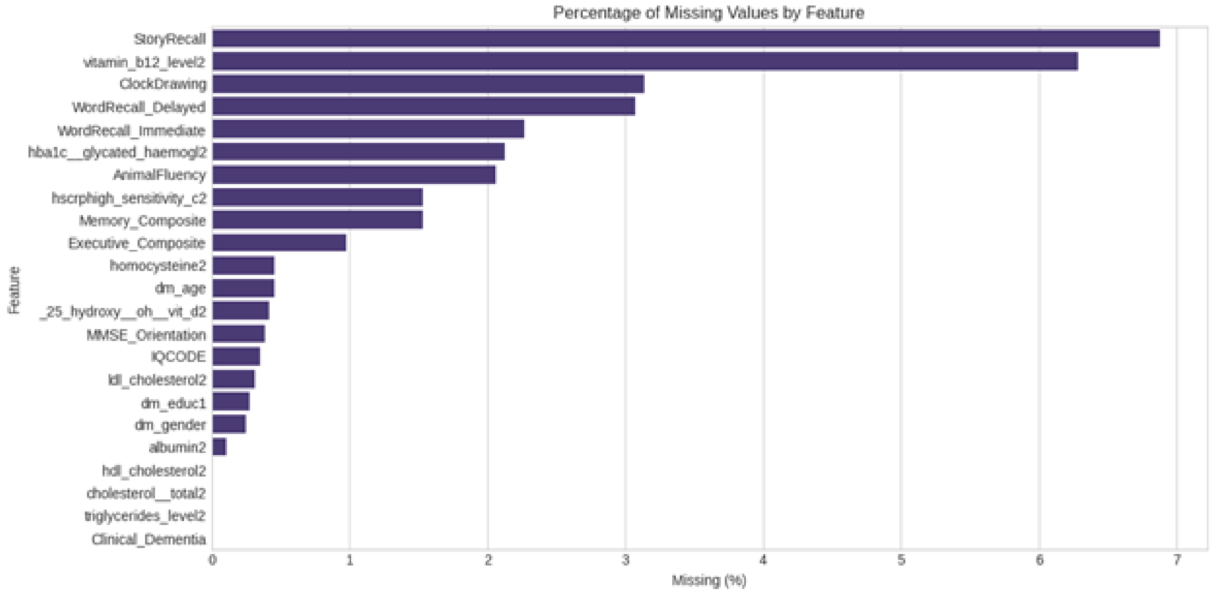

Supplementary Figure S3. Missingness across retained predictors prior to imputation.

Supplementary Figure S4.

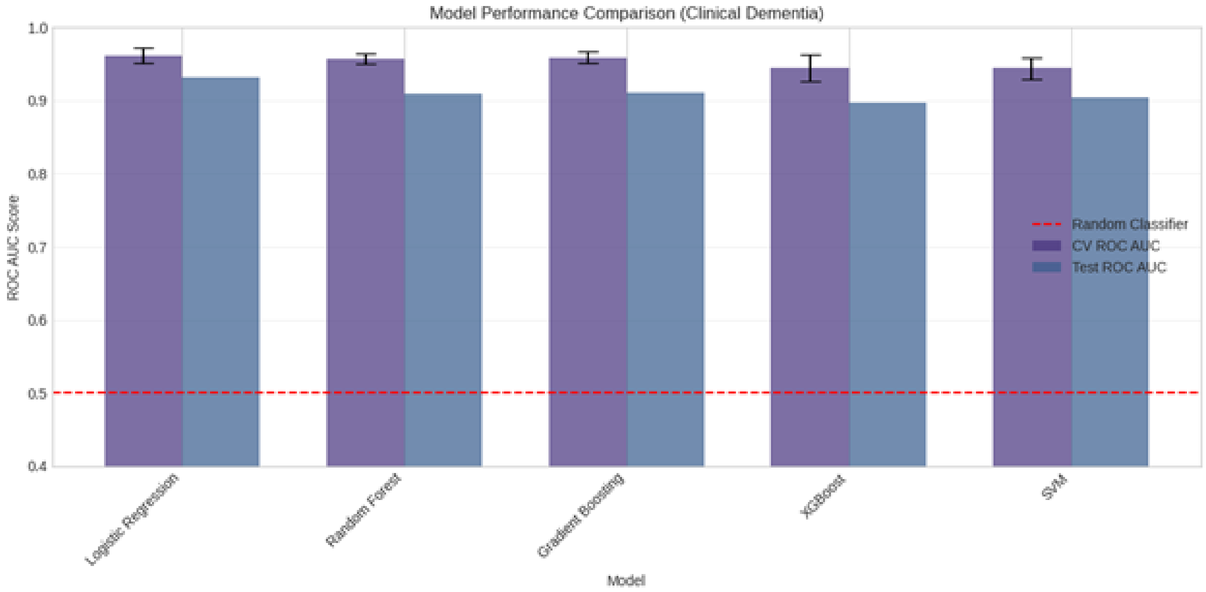

Supplementary Figure S4. Cross-validation and test-set ROC-AUC comparison across candidate models.

Supplementary Figure S5.

medRxiv preprint doi: https://doi.org/10.64898/2026.06.13.26354833; this version posted June 24, 2026. The copyright holder has placed this preprint (which was not certified by peer review) in the Public Domain. It is no longer restricted by copyright. Anyone can legally share, reuse, remix, or adapt this material for any purpose without crediting the original authors.

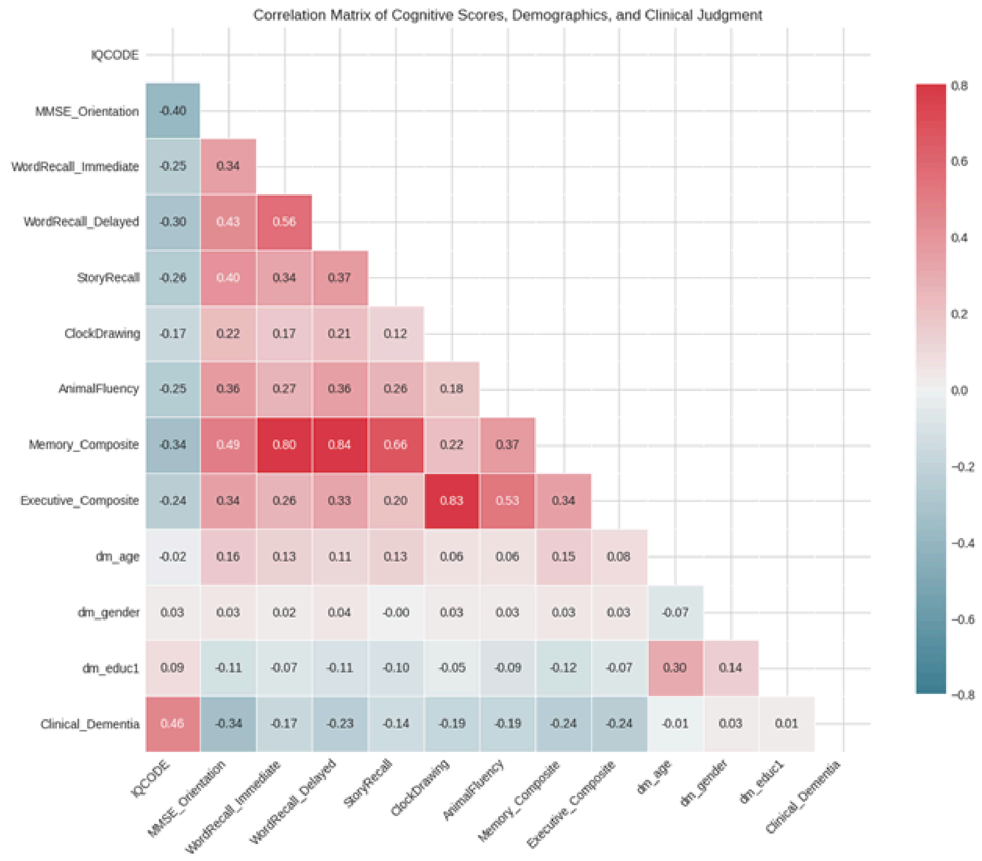

Supplementary Figure S5. Correlation matrix of core predictors.

Supplementary Figure S6.

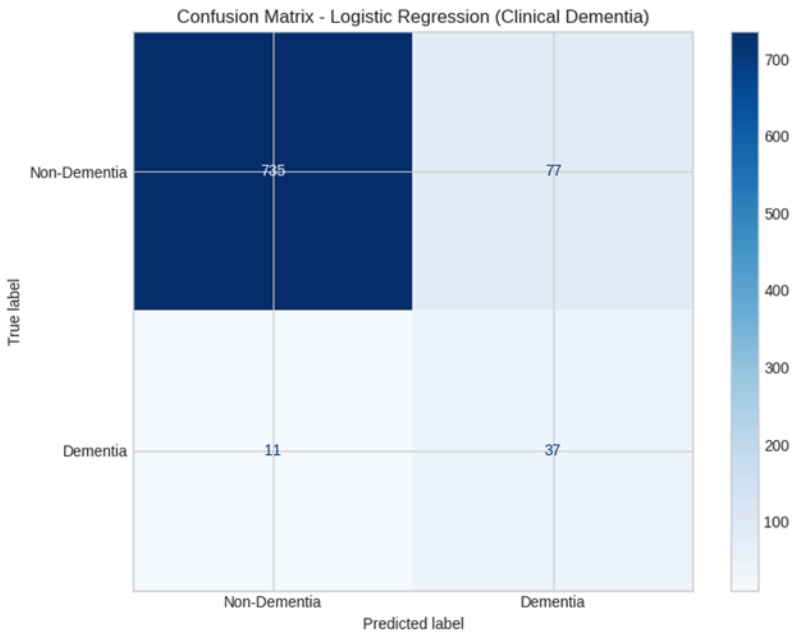

Supplementary Figure S6. Confusion matrix for final logistic regression model on the held-out test set.

medRxiv preprint doi: https://doi.org/10.64898/2026.06.13.26354833; this version posted June 24, 2026. The copyright holder has placed this preprint (which was not certified by peer review) in the Public Domain. It is no longer restricted by copyright. Anyone can legally share, reuse, remix, or adapt this material for any purpose without crediting the original authors.

Supplementary Figure S7.

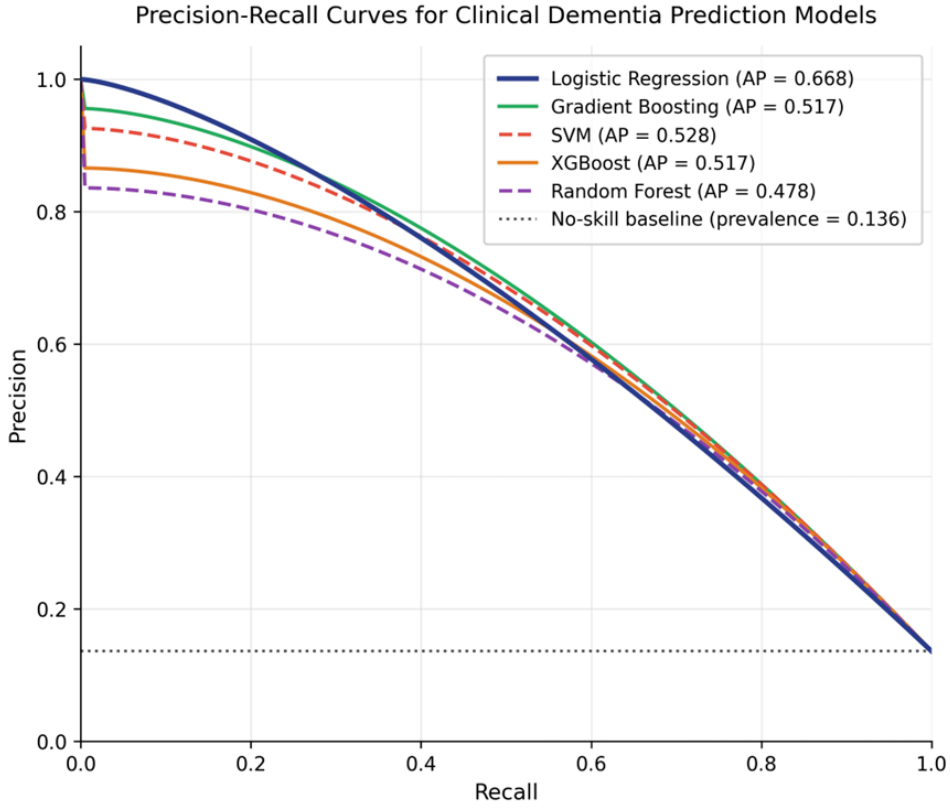

Supplementary Figure S7. Precision-recall curves for candidate dementia classification models on the held-out test set. Dashed line indicates no-skill baseline prevalence (0.136).

Supplementary Table S1. *Predictors included in the final model.*

**Domain**	**Predictor**
Informant-reported decline	IQCODE
Cognitive performance	MMSE orientation
Cognitive performance	Immediate word recall
Cognitive performance	Delayed word recall
Cognitive performance	Story recall
Cognitive performance	Clock drawing
Cognitive performance	Animal fluency
Cognitive performance	Memory composite

medRxiv preprint doi: https://doi.org/10.64898/2026.06.13.26354833; this version posted June 24, 2026. The copyright holder has placed this preprint (which was not certified by peer review) in the Public Domain. It is no longer restricted by copyright. Anyone can legally share, reuse, remix, or adapt this material for any purpose without crediting the original authors.

Cognitive performance	Executive composite
Cardiometabolic biomarker	Glycated haemoglobin (HbA1c)
Cardiometabolic biomarker	Total cholesterol
Cardiometabolic biomarker	HDL cholesterol
Cardiometabolic biomarker	LDL cholesterol
Cardiometabolic biomarker	Triglycerides
Cardiometabolic biomarker	High-sensitivity C-reactive protein (hsCRP)
Cardiometabolic biomarker	Homocysteine
Cardiometabolic biomarker	Vitamin B12
Cardiometabolic biomarker	25-hydroxy vitamin D
Cardiometabolic biomarker	Albumin
Sociodemographic characteristic	Age
Sociodemographic characteristic	Sex
Sociodemographic characteristic	Educational attainment

Supplementary Table S2. *Observed dementia prevalence across model derived risk categories.*

Risk category	Probability range	Estimated N	Estimated dementia cases	Observed prevalence (%)
Very Low	0.00-0.20	~ 2,565	~ 10	0.4
Low	0.21-0.40	~ 150	~ 7	4.4
Moderate	0.41-0.60	~ 105	~ 9	8.5
High	0.61-0.80	~ 105	~ 23	22.1
Very High	0.81-1.00	~ 261	~ 131	50.0
**Total**		**3,186**	**433**	**13.6**
